# Preimplantation Genetic Testing for Monogenic Disorders

**DOI:** 10.3390/genes11080871

**Published:** 2020-07-31

**Authors:** Martine De Rycke, Veerle Berckmoes

**Affiliations:** Center for Medical Genetics, Universitair Ziekenhuis Brussel, Laarbeeklaan 101, 1090 Brussels, Belgium; veerle.berckmoes@uzbrussel.be

**Keywords:** preimplantation genetic testing, monogenic disease, multiplex PCR, SNP array, NGS

## Abstract

Preimplantation genetic testing (PGT) has evolved into a well-established alternative to invasive prenatal diagnosis, even though genetic testing of single or few cells is quite challenging. PGT-M is in theory available for any monogenic disorder for which the disease-causing locus has been unequivocally identified. In practice, the list of indications for which PGT is allowed may vary substantially from country to country, depending on PGT regulation. Technically, the switch from multiplex PCR to robust generic workflows with whole genome amplification followed by SNP array or NGS represents a major improvement of the last decade: the waiting time for the couples has been substantially reduced since the customized preclinical workup can be omitted and the workload for the laboratories has decreased. Another evolution is that the generic methods now allow for concurrent analysis of PGT-M and PGT-A. As innovative algorithms are being developed and the cost of sequencing continues to decline, the field of PGT moves forward to a sequencing-based, all-in-one solution for PGT-M, PGT-SR, and PGT-A. This will generate a vast amount of complex genetic data entailing new challenges for genetic counseling. In this review, we summarize the state-of-the-art for PGT-M and reflect on its future.

## 1. Introduction

Preimplantation genetic testing (PGT) can be performed for monogenic disorders or single gene defects (PGT-M), for chromosomal structural rearrangements (PGT-SR), and for aneuploidy detection (PGT-A) [1]. PGT involves the biopsy of a single or few cells from in vitro fertilized embryos and testing of the biopsied samples for genetic aberrations followed by the selective transfer of embryos unaffected for the condition under study. Although genetic testing of single or few cells is challenging and the overall procedure is quite complex, PGT has evolved from an experimental procedure in the early 1990s to a well-established alternative to invasive prenatal diagnosis and possible therapeutic termination of pregnancy. The first report on children born after PGT was published by Handyside and colleagues in 1990, describing the use of PCR amplification for the detection of repetitive Y-sequences for gender determination in families with X-linked diseases [2]. The single cell simplex PCR applied in this earliest PGT-M approach was soon replaced by multiplex PCR testing, in which closely linked informative short tandem repeat (STR) markers are co-amplified, with or without the pathogenic variant amplicon. Single cell biopsy at day 3 followed by multiplex PCR became the method of choice for the detection of monogenic disorders [3]. Biopsy at the blastocyst stage followed by genome-wide technologies began to replace this gold-standard method over the last decade. The genome-wide methods yield data on genotyping as well as on chromosome copy number, allowing for concurrent analysis of PGT-M and PGT-A [4,5,6,7,8].

As PGT and especially PGT-M is technically complex, transport PGT was implemented, a procedure in which embryo samples biopsied in a satellite IVF laboratory are transported to a genetics unit for testing. Transport PGT has the advantage that testing can be performed by experienced teams in genetic laboratories specialized in single cell molecular diagnostics. Transport PGT is a service that has expanded substantially, despite the challenges related to transport and collaborations over (long) distances [9].

PGT guidelines and recommendations for good practice have been designed by several international societies such as the PGD International Society (PGDIS), the American Society for Reproductive Medicine (ASRM), and the European Society for Human Reproduction and Embryology (ESHRE) PGT Consortium. The latter society has recently updated and extended four sets of recommendations, covering guidance on the organization of PGT service as well as technical guidance on embryo biopsy and genetic testing [9,10,11,12].

In this review, we present an overview of current PGT-M practice from patient inclusion to baby follow-up and reflect on future developments.

## 2. Indications for PGT-M

The provision of PGT is legally restricted in many countries, yet, policies and regulations differ [13]. Some countries have quite restrictive laws with a clear line between acceptable and unacceptable indications with specific mechanisms in place for delineating which indications are eligible for PGT. For instance, the Human Fertilization and Embryology Authority oversees the acceptable use of PGT in the UK. In France, l’Agence de la Biomédecine is charged with the regulation of PGT. In other countries, the law is more liberal. A minority of countries have no government regulation. The USA, for example, has no established restrictions on PGT practice and as such, PGT is also used for nonmedical reasons such as social sexing [14]. A recent overview of regulatory frameworks in 43 European countries shows only two countries where PGT is not allowed (Malta and Bosnia & Herzegovina) [15]. The main concern raised from the dawn of PGT has been the fear for eugenics. Many countries have a legislation banning any form of eugenic selection, allowing to select against high risk and serious disorders in PGT-M and PGT-SR but excluding the selection or enhancement of non-pathological characteristics in humans. PGT-A fails to meet the standard of a ‘high risk and serious disorder’ and is therefore not permitted in 11 out of 43 European countries [15]. As the field is rapidly progressing, it is essential to continue the scientific, ethical, and legal debate about embryo selection and to make amendments when necessary. For instance, the implementation of preconception carrier screening is likely to bring more requests for double or triple conditions, which may not all meet the ‘high risk and serious disorder’ standard. It is clear that this should be balanced with thorough discussions and ethical reflections [16]. PGT-M can in theory be offered for all (combinations of) monogenic disorders for which the disease-causing loci have been unequivocally identified. These loci are nuclear (X-linked, autosomal, dominantly or recessively inherited) or mitochondrial (maternally inherited) and involve (likely) pathogenic genetic variant(s) (class 4–5) [17].

The requests can be for rare or for more common diseases. The more frequent indications for which PGT-M are currently applied are cystic fibrosis and hereditary hemoglobinopathies for the autosomal recessive disorders, and myotonic dystrophy type 1, neurofibromatosis, Huntington’s disease, and hereditary cancer syndromes for the autosomal dominant disorders. For the X-linked disorders, PGT is mainly carried out for Duchenne’s muscular dystrophy, hemophilia, and fragile X syndrome (unpublished data from the ESHRE PGT consortium). The advantages of specific DNA diagnosis over sexing for recessive X-linked disorders are twofold: healthy male embryos are not discarded and female carrier embryos can be identified and possibly used for transfer, according to the patient’s wishes and the center’s policy. PGT with sex selection for non-medical reasons such as family balancing is prohibited in most countries.

Some special indications have raised further ethical concerns. For instance, Human Leucocyte Antigen (HLA) matching of preimplantation embryos is an exceptional indication as it is not a pathological condition. PGT is applied to select an embryo that is HLA compatible with an affected sibling who will need a bone marrow transplantation in the future. Hematopoietic stem cells from the cord blood at birth or later from bone marrow of the PGT baby are used to transplant and cure the affected sibling. HLA typing alone is carried out for couples having a child with an acquired hematological malignancy, but mostly, HLA typing is combined with PGT for a monogenic disorder, commonly immunodeficiencies and hemoglobinopathies [18]. The selection of HLA-matched embryos has evoked many ethical debates. The possible instrumentalization of the child to be born is the main issue raised in these discussions. As a result, the regulation of PGT and HLA matching varies in different countries around the world. Local and national legislation usually allow the use of PGT to avoid the transmission of diseases for which no treatment exists, but only a subset of frameworks is permissive for PGT and HLA typing.

In families with a history of late-onset neurodegenerative disorders such as Huntington’s disease, individuals at risk who want to avoid pre-symptomatic testing but wish for their own biological unaffected children may opt for PGT with exclusion testing [19]. Exclusion testing recognizes the right of the parent not to know whether they are themselves affected while enabling them to have children not affected by the disease. Genetic implication counseling is a necessary part of the procedure [11]. Exclusion testing is indirect, based only on genetic markers, and involves the transfer of embryos carrying the haplotype derived from the non-affected grandparent. Embryos which have inherited the haplotype of the affected grandparent will be discarded as they have a 50% chance of being affected. These embryos have as well a 50% chance of being healthy for the disease under study. This fact together with the fact that about half of the couples will have an unnecessary IVF/PGT treatment with exposure to side effects and risks for the female and embryo, may be considered unethical. PGT with exclusion testing is therefore prohibited in some countries. The alternative with direct testing and non-disclosure of the results is not recommended as it obligates extreme confidentiality and may impose unethical behavior on the practitioners (for instance, fake embryo transfers) [20].

PGT for mitochondrial (mt) DNA pathogenic variants is offered in only few centers worldwide. The majority of mitochondrial DNA (mtDNA) pathogenic variants implicated in diseases show heteroplasmy, which is the co-existence of wildtype and pathogenic variant mtDNA in a single cell. The mtDNA pathogenic variant load (proportion of pathogenic variant mtDNA) may vary over time and differ from one cell type to another. Clinical symptoms manifest once a particular pathogenic variant load threshold has been exceeded. Because of a genetic bottleneck during oogenesis, the proportion of pathogenic variant mtDNA inherited from one generation to the next varies widely. PGT can be applied to select for embryos with a mtDNA pathogenic variant load below the threshold of clinical expression. It is an ethically difficult indication group as the approach reduces the risk for an affected child rather than eliminating it and this requires case-by-case counseling [21].

## 3. Genetic and Reproductive Counseling—Preclinical Workup

Before starting a clinical cycle, extensive genetic and reproductive counseling is provided to the prospective parent(s). Psychological support may be offered as well. Parents are asked to sign informed consent and blood samples are collected for preclinical workup. For PGT-M this usually includes blood samples and genetic reports from relevant first-degree family members. The preclinical reproductive workup and ovarian stimulation is similar as for patients undergoing conventional IVF. The preclinical genetic workup requires a conventional karyotype of both partners. This can be complemented by screening tests for carriership of common genetic variants for cystic fibrosis, spinomuscular atrophy, or hemoglobinopathies. In the near future, these individual screening tests will be most likely replaced by extended carrier screening. Genetic reports should be available at the intake of a PGT request. The preclinical genetic workup for the monogenic disorder depends on the test methodology (targeted versus genome-wide testing) and the strategy (an indirect test based on genetic markers versus a direct test including the detection of the pathogenic variant).

## 4. IVF, Embryo Biopsy, Transfer, and Cryopreservation

### 4.1. IVF and Current Embryo Biopsy Methods

Fertilization by ICSI rather than regular IVF is recommended for PGT treatment, in order to avoid contamination from remaining cumulus cells or residual sperm cells attached to the zona pellucida. Certain PGT indications may be associated with reduced spermatogenesis, and present with lowered fertilization rates. For instance, it has been known that the *PKD1* and *PKD2* genes underlying autosomal dominant polycystic kidney disease (ADPKD) play a role in the male reproductive system. Males affected with ADPKD may present with lower sperm motility and lower sperm concentration. A recent study showed that fertilization rates and live birth delivery rates tended to be lower for couples with the male partner affected with ADPKD, compared to couples with the female partner affected with ADPKD, although the higher female age in the former group was a confounding factor [22]. In some centers, oocyte or embryo vitrification before biopsy is applied as a systematic approach to accumulate a larger number of embryos for testing while in other cases it is applied as a rescue strategy in a minority of cycles, when in need for rapid interventions to preserve fertility such as in cases of cancer treatment [23,24,25].

Biopsy can be performed at different developmental stages. All present methods are invasive. Biopsy of the first and second polar body (both are required for an accurate diagnosis) is currently applied in only a minority of centers (unpublished data from the ESHRE PGT consortium of 2016 and 2017). An advantage is that the removal of polar bodies has no detrimental effect on embryonic development, however the most important limitation is that only the maternal genetic contribution can be evaluated.

Cleavage-stage embryo biopsy has been the gold-standard for many years. It implies zona opening (mechanically, chemically, or by using laser energy) and blastomere removal, mainly by aspiration, on day 3 of preimplantation development. Major disadvantages of day 3 biopsy are the limited amount of DNA available for testing and the negative impact of the removal of embryonic cells. It was shown that a two-cell removal at the cleavage stage harms embryonic development and implantation potential more than the removal of one cell [26]. Therefore, the removal of a single cell at the cleavage stage has been recommended. Cleavage-stage biopsy leaves sufficient time for genetic analysis before fresh embryo transfer on day 5. If available, supernumerary genetically transferable embryos can be cryopreserved for later use.

Blastocyst or trophectoderm (TE) biopsy is at present the most widely used technique [27]. Laser energy is used to open the zona pellucida, either on day 3/4 or on day 5. TE cells are aspirated and excised with a laser from herniating blastocysts, or aspirated in combination with mechanical dissection from blastocysts, usually on day 5/6. TE biopsy provides more cells (ideally five to eight cells) for genetic analysis providing a better accuracy [28]. This embryonic stage is also considered less sensitive to possible embryo damage as the inner cell mass from which the fetus originates is left intact. A paired clinical trial showed that implantation rates of 50% in the nonbiopsied group were diminished to 30% in the cleavage-stage biopsy group, while similar implantation rates were obtained for the blastocyst-biopsy group versus the nonbiopsied group [29]. Another benefit of TE biopsy is the lower level of chromosomal mosaicism at this stage as compared to the cleavage stage. The problem of limited time for analysis in the case of a fresh embryo transfer at day 5/6 is overcome by vitrification and embryo transfer in a deferred cycle.

### 4.2. Current Developments and Future Sampling Methods

An alternative method is morula-stage biopsy, performed on day 4, after artificial decompaction using Ca/Mg-free medium [30]. This option is attractive as more cells are obtained compared with cleavage-stage biopsy, cells are intact in contrast to TE biopsy where cells may be damaged and fresh embryo transfer is still possible. A drawback is the inability to distinguish between inner cell mass and TE cells. Whether or not the removal of several cells at day 4 has a negative impact on embryo development and implantation remains largely unknown. Irani and colleagues relied on morula biopsy at day 6 for slowly developing embryos in order to enlarge the cohort of available embryos for testing. Of note were findings of lower implantation and live birth rates as well as higher rates of complex aneuploidy [31].

Another option is blastocentesis, which is the aspiration of blastocoel fluid (BF) containing cell-free DNA (cfDNA) from the blastocoel cavity with a fine needle [32]. This procedure is considered less invasive than TE biopsy and results in embryo collapse which is a manipulation applied in routine vitrification practice to maximize embryo survival post-vitrification [33]. A true non-invasive alternative is to rely on cfDNA present in the spent blastocyst medium (SBM) [34]. Several studies showed that the karyotype concordance between blastocoel fluid samples and inner cell mass and/or TE cells varied widely, indicating that genetic analysis following blastocentesis is insufficiently accurate for clinical PGT-A/PGT-SR [35]. Moreover, the diagnostic efficiency was low with high amplification failure rates, making blastocentesis also unsuitable for PGT-M.

Analysis of cfDNA of spent embryo culture medium collected at the blastocyst stage seems a more promising sampling method. A recent study of Capalbo and colleagues compared blastocoel fluid and spent culture medium samples with TE cells as template for PGT-M [35]. TE samples showed 100% amplification and a high genotype concordance rate (99.8%). Blastocoel fluid samples gave high amplification failure (72.6%) with low genotype concordance (13.3%). SBM samples performed better with low amplification failure (10.3%) and an intermediate accordance rate of 59.5%. The lower diagnostic accuracy rate was due to contamination derived from maternal DNA due to incomplete oocyte denudation and from exogenous DNA present in supplements of culture media. As contamination is a major risk factor for genetic misdiagnosis, it is necessary to further optimize current protocols to favor embryo-specific analysis and allow discrimination between embryonic and non-embryonic DNA. This will provide more reliable and accurate testing for PGT-M in the short-term. Future research should also focus on the origin of the cell-free DNA to determine whether collected samples reflect the real genetic status of the embryo [36]. Substituting the laser-based biopsy methods by non-invasive sampling would definitely transform the field of PGT as it represents a safer option for the embryo and it would make the overall treatment less expensive.

### 4.3. Embryo Transfer and Cryopreservation

More and more PGT centers carry out a single embryo transfer (SET), a policy that can be linked with mechanisms of reimbursement and legislation, but also with the acknowledgement that SET is associated with a safer clinical outcome for any ensuing pregnancy. The substitution of slow-freezing by vitrification greatly contributed to the widespread use of SET. Vitrification has been shown to be superior to slow-freezing in terms of survival rate for both cleavage-stage embryos and blastocysts [37]. The evidence overall indicates that clinical outcomes after elective frozen embryo transfer are equivalent with the outcome after fresh embryo transfer for the general population (normo-ovulatory patients) and prove even better for specific subgroups such as patients at high risk of ovarian hyperstimulation syndrome (OHSS) [38].

The implementation of vitrification also changed the overall timeline of a PGT cycle. For many years, embryo biopsy on day 3, and testing and embryo transfer on day 5/6 were carried out within the timeframe of one cycle; the transfer was a ‘fresh embryo transfer’ and any surplus embryo from that cycle was cryopreserved. An alternative timeline emerged for cases for which no fresh embryo transfer was possible (high risk of OHSS or endometrium problems). During this freeze-all strategy, all genetically suitable embryos are vitrified and transfer was scheduled in a later cycle. Current comprehensive genetic testing is mostly linked with TE biopsy and a freeze-all strategy. This enables a more efficient and cost-effective laboratory organization as the larger time windows make it possible to pool and co-process samples of multiple patients. Once the genetic testing results have been disclosed, only genetically suitable embryos remain cryopreserved.

## 5. Diagnostic Methods

### 5.1. Early Methods of PGT-M

**Figure 1:**
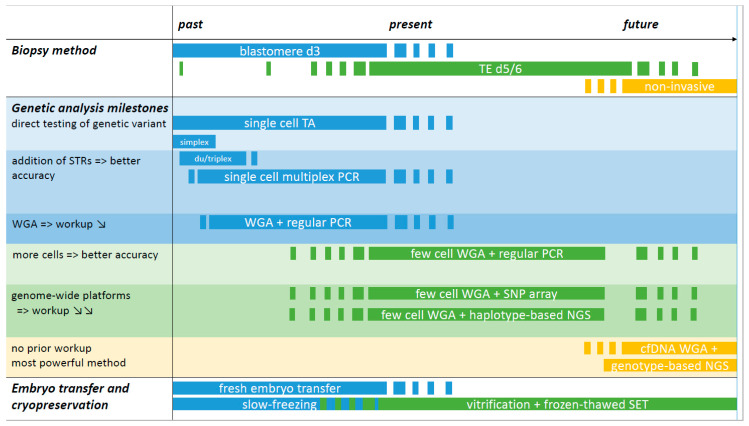
Overview of past, present, and future methods for PGT-M. The timeline is divided over past, present, and future sections without precise timepoints and starts in the early 1990s with blastomere biopsy and simplex PCR amplification for detection of the genetic variant in PGT-M. If the amplicon did not generate a difference in fragment length, post-PCR reactions were applied to allow low- and high-risk allele discrimination. These methods are currently still employed, for instance for detection of (de novo) genetic variants following WGA and SNP array. The addition of STR markers flanking the region of interest increased the accuracy of the PCR test: with every informative marker added, the diagnosis is confirmed and contamination and ADO pitfalls can be detected. The use of commercial PCR multiplex kits facilitated the development of single cell PCR reactions and duplex or triplex PCRs became multiplexes of 10 or more amplicons. Single cell multiplex PCR has been the gold standard for over two decades, alongside blastomere biopsy at day 3 and fresh embryo transfer on day 5/6. TE biopsy is currently the norm for embryo biopsy and is linked with the freeze-all strategy. The substitution of slow-freezing by vitrification greatly contributed to the widespread use of SET. WGA represents a technical milestone. The method of single or few cell WGA followed by regular PCR of multiple STRs with or without the genetic variant amplicon is a more universal method with a reduced workup as the adaptation/validation of PCR reactions to the level of single or few cells can be omitted. The implementation of WGA followed by genome-wide SNP array or NGS represents a truly generic method, making it possible to combine haplotyping results for PGT-M with genome-wide chromosome copy number PGT-A data. Both platforms require a sample of a valid reference family member for haplotyping. Genotype-based NGS is regarded as the most powerful platform for future PGT as it will allow an all-in-one solution for direct genotyping and chromosome aberration assessment. Whether TE biopsy will be replaced by non-invasive sampling methods for PGT in the future requires further investigations. TA: targeted amplification: i.e., PCR-based amplification of the region of interest, either the genetic variant and/or a genetic marker(s); WGA: whole genome amplification: WGA products are used in downstream amplification reactions, either TA (regular PCR) or via genome-wide platforms (SNP array or NGS); STR: short tandem repeat marker; SNP: single nucleotide polymorphism marker; TE: trophectoderm; cfDNA: cell-free DNA; SET: single embryo transfer.

An overview of past, present, and future methods for PGT-M is summarized in Figure 1. At the start of PGT-M in the early 1990s, single cell simplex PCR amplification was applied. From these earliest approaches, contamination and allele drop out (ADO) surfaced as important issues that could lead to misdiagnosis [39]. Contamination with extraneous DNA or carry-over from previous amplification reactions was and still is a major problem, given the high number of amplification cycles that is required to increase the minute amount of DNA. It can be minimized by taking rigorous prevention measures. ADO originates from the unequal amplification of alleles present in a heterozygous sample (called preferential amplification) to the point where an allele remains undetected. Control for ADO relies on optimized methods for cell lysis and amplification and on sensitive methods for allele detection. By far, the most important measure to control and detect ADO as well as contamination was the inclusion of closely linked informative short tandem repeat (STR) markers in the PCR reaction. The co-amplification of STR markers with or without the pathogenic variant amplicon(s) at the level of a single or few cells yields a more accurate test and this so-called haplotyping approach has been the gold-standard for over two decades [27,40]. A major limitation of this approach is that the development and validation of a single cell multiplex PCR is labor intensive for the laboratory and couples face a long waiting time.

### 5.2. Whole Genome Amplification Approaches

The implementation of single or few cell whole genome amplification (WGA) was a technical improvement which stimulated the development of more generic approaches. The first WGA methods were PCR-based and suffered from incomplete genome coverage and amplification bias. The use of *Taq* DNA polymerase yielded an average fragment length of 400–500 bp (with a maximum size of 3 kb) and introduced many DNA sequence errors [41]. A multiple displacement amplification (MDA) method relying on isothermal strand displacement amplification was developed at the single cell level more than a decade ago [41]. In a MDA reaction, random exonuclease-resistant primers anneal to the denatured target DNA and a DNA polymerase with strand-displacement activity such as *Phi29* elongating the primers in an isothermal reaction at 30 °C. Additional priming events can occur on each displaced strand leading to a network of branched DNA strands over 10 kb. Because of the proofreading activity of the *Phi29* polymerase, the error rate of MDA-based WGA is much lower compared with *Taq* DNA polymerase-based methods, but the non-linear amplification yields over- or under-representation of genomic regions [42]. Afterwards, WGA methods combining MDA and PCR amplification were introduced. Both the Rubicon PicoPLEX and the MALBAC (Multiple Annealing and Looping Based Amplification Cycles) protocol initiate with DNA fragmentation and a pre-amplification MDA reaction using hybrid primers, followed by PCR [43,44].

None of the WGA methods are producing a true linear representation of the single or few cell genome and the results vary in ADO, preferential amplification rate, coverage, and nucleotide copy errors. As a consequence, a specific WGA method is chosen in function of the downstream application: the Rubicon PicoPLEX protocol is currently the method of choice for the detection of chromosomal copy number because of the reduced amplification bias while MDA is preferred for haplotyping applications in case of monogenic disorders because of the better genome coverage and low error rates [45,46,47]. Final data interpretation has to take into account bias and artefacts introduced from WGA. Also artefacts from the cell cycle phase should be considered. Analysis of TE samples containing cells in different cell cycle stages may overcome this problem while the WGA representation bias may be partially filtered out by computational algorithms but can not be completely eliminated.

WGA followed by standard PCR reactions of a multitude of STR markers flanking the region(s) of interest is simpler and requires a shorter validation, compared to single or few cell multiplex PCR [48]. WGA followed by genome-wide methods based on single nucleotide polymorphism (SNP) array or next-generation sequencing (NGS), represents a truly comprehensive approach.

### 5.3. SNP Array for PGT-M

SNP arrays are high density oligo arrays containing up to several million probes, which allow genotyping of hundreds of thousands of selected SNPs across all chromosomes in a single reaction. SNPs are mostly biallelic, alleles are indicated as A and B and genotypes are homozygous AA or BB, or heterozygous AB. The commercially available SNP arrays use different methods for SNP genotyping: hybridization to SNP allele-specific probes or single base extension reactions are often applied [49]. The arrays are scanned and SNP genotypes are called based on the total fluorescence and the ratio of hybridization intensities for A and B (allele frequencies) (for example AB is called in case of similar intensities of an intermediate level). Targeted multiplex PCR and SNP array share the same principle of linkage-based testing for PGT-M, but the SNP array workflow is much more standardized and uniform, without the need for a locus-specific preclinical workup. This reduces the laboratory workload and the waiting time for the couples substantially. A drawback of SNP arrays is the high cost of equipment and consumables which seems to hamper their widespread clinical use. The SNP array platform is especially powerful for double indications (for instance two monogenic disorders or a monogenic disorder plus HLA matching) as whole genome haplotyping is accomplished from a single data set. Haplotyping via SNP array can also be applied for balanced translocations or inversions. Analysis reaches a high resolution and can distinguish normal from balanced translocation carriers. The major requirements for SNP array application are that the chromosomal or monogenic aberration(s) is/are inherited and relevant family samples are available for haplotyping.

Different SNP genotyping algorithms are available. Handyside and coworkers developed karyomapping, a family-based computational phasing approach for reconstruction of SNP haplotypes which flank the pathogenic variant(s) [5,50]. It is applicable for both SNP arrays and NGS. The commercially available karyomapping algorithm uses discrete diploid SNP calls (assuming AA, BB, AB, or No call as possible states for each SNP) together with basic Mendelian laws and requires a close relative for phasing.

### 5.4. PGT-M for De Novo Pathogenic Variants

In case of a de novo pathogenic variant(s) or when relevant DNA samples of family members cannot be obtained, it is necessary to include the genetic variant detection in the test strategy. This is feasible in targeted—as well as in genome-wide—methods, but the validation step of the latter methods is much easier. Many types of genetic variants can be detected by PCR supplemented with a post-PCR reaction if necessary. Deletion or duplication variants of a few nucleotides can be detected directly via fragment length difference. For single nucleotide substitutions, different strategies of allele discrimination have been developed, e.g., minisequencing [51]. The direct detection of complex and/or larger gene rearrangements has so far been difficult since the exact breakpoints of the rearrangement are frequently unknown. High-resolution characterization of breakpoints located outside highly repetitive regions is now achievable with long-read nanopore sequencing [52].

Since high-risk and low-risk haplotypes cannot be established during workup, segregation must be determined and diagnosis can be obtained during the first PGT cycle(s), provided that sufficient embryos are available with at least one embryo diagnosed as affected and with the pathogenic variant consistently detected in the presence of the same parental haplotype.

### 5.5. SNP Array for Concurrent PGT-M and PGT-A

SNP array can allow for simultaneous analysis of PGT-M and PGT-A as both SNP genotype and chromosome copy number info are obtained from the raw data set. As such, SNP arrays can reveal the presence of aneuploidies, polyploidies, and uniparental disomy [53]. Two measures provide evidence about the copy number state: the log R ratio (the log2 transformed value of the normalized intensity of the SNP) and the B allele frequency (BAF, which is the signal intensity of the B allele over the total signal intensity for a SNP). BAF values of 0, 0.5, and 1 represent a normal copy number (n = 2) but aberrations will cause a decrease or increase of the total intensity and allele frequencies. SNP arrays at the level of a single or few cells yield a lot of noise because of WGA pitfalls and therefore demand particularly well-developed algorithms for data interpretation. Genotyping algorithms using discrete diploid SNP calls such as karyomapping will yield errors across regions with copy number variations (true or WGA-induced) and therefore have restrictions in the detection of copy number aberrations. So far, few well-developed algorithms providing an all-in-one solution for data interpretation have been published. Some PGT centers rely on in-house developed extended or novel algorithms, to overcome this limitation. For instance, haplarithmisis is a computational pipeline that primarily relies on continuous BAFs and allows haplotype and copy number detection as well as determination of the parental origin of the chromosomal anomaly [6].

The true clinical benefit of PGT-A remains a topic of discussion, neither is there a consensus about adding PGT-A routinely to PGT-M cases, yet, concurrent PGT-A and PGT-M is increasingly regarded as an acceptable option, since it does not entail additional procedures or higher risks for the couple or future child.

### 5.6. NGS for Concurrent PGT-M and PGT-A

NGS involves DNA fragmentation and preparation of a library of templates using adapters containing barcodes for a more affordable analysis with multiple samples in a single run. The single molecule templates are then sequenced in parallel from one end or from both ends, either directly (third-generation) or after prior clonal amplification (second-generation) and the sequence reads are mapped to a reference genome. A crucial parameter is the genome coverage or read depth referring to the number of reads that is found at a given genomic position. A relatively low average coverage has been demonstrated as sufficient for accurate numerical chromosome analysis [54]. For monogenic disorders, sequencing at high coverage is required, which at this time is still too expensive for routine clinical applications at a whole-genome scale, and therefore, various strategies, aiming at affordable and rapid protocols are being developed. These protocols often provide a tandem solution, combining PGT-A with PGT-M, and can be classified in two groups, depending on whether sequencing data for the monogenic locus are derived with a targeted or a genome-wide method.

The three approaches of targeted sequencing presented here are merely an illustration of the wide range of possibilities. A more affordable solution is to increase the read depth across the site of the pathogenic variant (minimum 100×) only. In one report, the pathogenic variant loci were captured in a preamplification reaction with pathogenic variant specific primers while the concurrent inclusion of a specially designed primer pool allowed for parallel aneuploidy screening via real-time quantitative PCR [4]. The MARSALA method (mutated allele revealed by sequencing with aneuploidy and linkage analyses) works in a similar way: an aliquot of MALBAC-based WGA products undergoes targeted amplification and the mixture of WGA and targeted enriched templates is subsequently sequenced at low depth (0.1–2×), yielding targeted SNP haplotyping results for PGT-M together with genome-wide PGT-A data [55]. Chamayou and colleagues developed a universal strategy for concurrent PGT-M for cystic fibrosis and PGT-A [56]. Part of the MDA-based WGA products undergo multiplex PCR for coverage of the *CFTR* gene (pathogenic variants and SNPs), generating a targeted DNA library. Another aliquot of the WGA products is processed for comprehensive chromosome analysis, generating a genome-wide DNA library. Both DNA libraries are subsequently sequenced. In these three examples, the targeted amplification is coupled with the need for a locus-specific preclinical workup.

Genome-wide NGS is regarded as the most powerful platform for future PGT, as it will offer simultaneously genotype and chromosome copy number data with increased accuracy, reliability, and resolution, allowing a generic protocol for monogenic disorders (including the detection of de novo pathogenic variants and repeat expansions) and numerical and structural chromosomal aberrations (including balanced rearrangements). As the current cost is too high, the main approach has been to decrease the number of reads by reducing the complexity of the libraries. Some of these strategies are listed below (non-exhaustive).

The OnePGT solution, commercialized by Agilent, is a NGS-based generic application for PGT-M, PGT-SR, and PGT-A [8]. The method takes advantage of reduced-representation genome sequencing in offering a single workflow, starting from MDA-based WGA and followed by library preparation with double restriction enzyme digestion and enrichment for fragments in a specific range of length; sequencing data interpretation relies on the haplarithmisis algorithm with concurrent chromosome copy number detection and SNP linkage-based haplotyping. The PGT-M module requires a minimum of 1.6× coverage and cosequencing of samples from the parents and a valid reference family member.

Haploseek is another universal workflow offering an economical analysis of PGT-M and PGT-A within a 24-hour protocol, starting from PicoPLEX-based WGA and genome-wide low coverage sequencing (0.3–1.4×) [7]. The information of high quality whole genome haplotypes of the couple and a reference obtained through SNP array is then integrated with the sample sequencing data and a hidden Markov model is used to predict whether haplotypes of the samples are shared with the reference or not.

In another NGS approach, a target enrichment gene panel with nearly 5000 Mendelian disease-associated genes (TruSight One sequencing panel) was applied on MDA-based WGA products, thereby offering direct testing of family pathogenic variant(s) plus indirect pathogenic variant detection through haplotyping of SNPs together with chromosome copy number detection through the log ratio of signal intensities, i.e., PGT-M and PGT-A together in a single workflow [57].

It is clear that the implementation of concurrent PGT-M and PGT-A offers further possibilities but may also generate incidental findings and more complex genetic information which we currently do not fully understand. This entails many ethical discussions and challenges for genetic counseling, because we lack knowledge to support data interpretation.

## 6. Clinical Outcome

It is difficult to compare clinical outcomes between individual centers because of varying factors such as maternal age, indication type, or differences in genetic testing and IVF procedures. Proper benchmarking against consortia data is neither straightforward, since reports of large data collections have been lagging. The most recent publication is about summary PGT data for 2015 from the ESHRE European IVF-Monitoring Consortium. This data set, which had been collected from 23 countries, showed a pregnancy rate of 39.7% per fresh embryo transfer cycle and a rate of 41.0% per frozen embryo transfer, over all PGT indications [58].

A high diagnostic efficiency is a prerequisite for any laboratory wishing to perform PGT as this potentially increases the clinical outcome. This is particularly true for indications with limited success rate a priori, such as for PGT-M with HLA typing where the chance of finding a genetically compatible embryo was shown to be only 16% [59]. This yielded a live birth delivery rate of 30.3% per transfer, showing that PGT with HLA typing is a valuable procedure where the high complexity and limited delivery rate are balanced by the successful transplantation outcome and the positive impact on families.

Apart from technical errors due to ADO and contamination, possible causes of misdiagnosis involve human errors such as mislabeling or incorrect embryo transfer. The misdiagnosis rate as published by the ESHRE PGT consortium is generally very low (<0.1%) [27]. The true misdiagnosis rate is difficult to assess as many embryo transfers yield no pregnancy or birth and only a minority of children have pre- or postnatal testing. PGT centers are encouraged to re-analyse part of the non-transferred embryos to provide an in-house estimate of the misdiagnosis rate.

## 7. Children Follow-Up

It was hypothesized that assisted reproductive technologies, especially the more invasive techniques like ICSI, would increase the risk for birth defects. Studies showed that IVF and ICSI are associated with a small but statistically significant increase in congenital anomalies at birth, compared with the general population [60,61]. No difference was observed between ICSI and IVF [62].

Follow-up data on children conceived after PGT with ICSI and embryo biopsy procedures are still limited. Neonatal follow-up studies did not report a higher rate of major congenital malformations after PGT when compared to IVF/ICSI [63,64,65]. The study from our center compared neonatal data of 995 live born children after PGT with the outcome data of 1507 live born children after ICSI and showed that embryo biopsy at cleavage stage did not add a significant risk to the overall medical condition of newborn children nor did it change the risk for major malformations [63]. These results were in line with studies carried out in other centers using embryo biopsy at cleavage stage [64,65]. The recent retrospective study in which a cohort of 1,721 children born after PGT with blastocyst biopsy and cryopreservation was compared with an IVF/ICSI control group showed no significant difference in neonatal outcome, indicating that neither embryo biopsy at blastocyst stage nor cryopreservation added further risk to the health of PGT children [66].

The follow-up of PGT children at a young age has been studied in smaller cohorts. Developmental neurological and cognitive assessment and follow-up on psychomotor and social functioning showed that PGT pre-schoolers were comparable with controls born after ICSI or after spontaneous conception [67,68,69,70]. Another study on body composition and blood pressure showed no adverse outcomes for 6-year-old children born after PGT (with day 3 embryo biopsy followed by blastocyst transfer) compared to children born after ICSI without embryo biopsy [71]. In summary, the follow-up results have so far been reassuring but further monitoring of the safety of PGT and the long-term health of the children remains necessary.

## 8. Conclusions and Future Perspectives

Major advancements have been introduced in the area of PGT and assisted reproduction over the years, making PGT a well-established, accurate, and safe clinical procedure. The implementation of genome-wide methods has allowed more standardization and uniformity for the genetic laboratories. However, additional genetic findings other than the requested genetic condition—such as chromosomal mosaicism in embryos tested for a monogenic disorder— have posed new dilemmas for genetic counseling and embryo transfer policy making. As the cost of sequencing continues to decline, PGT moves technically towards a sequencing-based, all-in-one solution for PGT-M, PGT-SR, and PGT-A. As there is more awareness among patients about the risks of transmitting genetic disorders and since the number of diseases with identifiable genetic cause(s) continues to rise, the total number of treatments as well as the list of indications for PGT is likely to expand. Whether the scope of PGT indications will broaden in the future from diagnosis of monogenic disorders to also predicting the risk of polygenic disorders (PGT-P) represents an emerging ethical challenge for PGT practice. It is clear that the rapid technological advances should be balanced with ethical reflection and thorough discussions.

## References

[1] Zegers-Hochschild F., Adamson G.D., Dyer S., Racowsky C., de Mouzon J., Sokol R., Rienzi L., Sunde A., Schmidt L., Cooke I.D. (2017). The International Glossary on Infertility and Fertility Care, 2017. Hum. Reprod..

[2] Handyside A.H., Kontogianni E.H., Hardy K., Winston R.M. (1990). Pregnancies from biopsied human preimplantation embryos sexed by Y-specific DNA amplification. Nature.

[3] Laurie A.D., Hill A.M., Harraway J.R., Fellowes A.P., Phillipson G.T., Benny P.S., Smith M.P., George P.M. (2010). Preimplantation genetic diagnosis for hemophilia A using indirect linkage analysis and direct genotyping approaches. J. Thromb. Haemost..

[4] Treff N.R., Fedick A.M., Tao X., Devkota B., Taylor D., Scott R.T. (2013). Evaluation of targeted next-generation sequencing–based preimplantation genetic diagnosis of monogenic disease. Fertil. Steril..

[5] Natesan S.A., Bladon A.J., Coskun S., Qubbaj W., Prates R., Munné S., Coonen E., Dreesen J.C., Stevens S.J., Paulussen A.D. (2014). Genome-wide karyomapping accurately identifies the inheritance of single-gene defects in human preimplantation embryos in vitro. Genet. Med..

[6] Esteki M.Z., Dimitriadou E., Mateiu L., Melotte C., Van Der Aa N., Kumar P., Das R., Theunis K., Cheng J., Legius E. (2015). Concurrent Whole-Genome Haplotyping and Copy-Number Profiling of Single Cells. Am. J. Hum. Genet..

[7] Backenroth D., Zahdeh F., Kling Y., Peretz A., Rosen T., Kort D., Zeligson S., Dror T., Kirshberg S., Burak E. (2018). Haploseek: A 24-hour all-in-one method for preimplantation genetic diagnosis (PGD) of monogenic disease and aneuploidy. Genet. Med..

[8] Masset H., Esteki M.Z., Dimitriadou E., Dreesen J., Debrock S., Derhaag J., Derks K., Destouni A., Drüsedau M., Meekels J. (2019). Multi-centre evaluation of a comprehensive preimplantation genetic test through haplotyping-by-sequencing. Hum. Reprod..

[9] Carvalho F., Coonen E., Goossens V., Kokkali G., Rubio C., Meijer-Hoogeveen M., Moutou C., Vermeulen N., De Rycke M. (2020). ESHRE PGT Consortium good practice recommendations for the organisation of PGT. Hum. Reprod. Open.

[10] Kokkali G., Coticchio G., Bronet F., Celebi C., Cimadomo D., Goossens V., Liss J., Nunes S., Sfontouris I., Vermeulen N. (2020). ESHRE PGT Consortium and SIG Embryology good practice recommendations for polar body and embryo biopsy for PGT. Hum. Reprod. Open.

[11] Carvalho F., Moutou C., Dimitriadou E., Dreesen J., Giménez C., Goossens V., Kakourou G., Vermeulen N., Zuccarello D., De Rycke M. (2020). ESHRE PGT Consortium good practice recommendations for the detection of monogenic disorders. Hum. Reprod. Open.

[12] Coonen E., Rubio C., Christopikou D., Dimitriadou E., Gontar J., Goossens V., Maurer M., Spinella F., Vermeulen N., De Rycke M. (2020). ESHRE PGT Consortium good practice recommendations for the detection of structural and numerical chromosomal aberrations. Hum. Reprod. Open.

[13] Ginoza M.E.C., Isasi R. (2019). Regulating Preimplantation Genetic Testing across the World: A Comparison of International Policy and Ethical Perspectives. Cold Spring Harb. Perspect. Med..

[14] Bayefsky M.J. (2016). Comparative preimplantation genetic diagnosis policy in Europe and the USA and its implications for reproductive tourism. Reprod. Biomed. Soc. Online.

[15] Calhaz-Jorge C., De Geyter C.H., Kupka M.S., Wyns C., Mocanu E., Motrenko T., Scaravelli G., Smeenk J., Vidakovic S., Goossens V. (2020). Survey on ART and IUI: Legislation, regulation, funding and registries in European countries: The European IVF-monitoring Consortium (EIM) for the European Society of Human Reproduction and Embryology (ESHRE). Hum. Reprod. Open.

[16] Dondorp W., De Wert G. (2019). Refining the ethics of preimplantation genetic diagnosis: A plea for contextualized proportionality. Bioethics.

[17] Richards S., Aziz N., Bale S., Bick D., Das S., Gastier-Foster J., Grody W.W., Hegde M., Lyon E., Spector E. (2015). Standards and Guidelines for the Interpretation of Sequence Variants: A Joint Consensus Recommendation of the American College of Medical Genetics and Genomics and the Association for Molecular Pathology. Genet. Med..

[18] Kakourou G., Kahraman S., Ekmekci G.C., Tac H.A., Kourlaba G., Kourkouni E., Sanz A.C., Martin J., Malmgren H., Giménez C. (2018). The clinical utility of PGD with HLA matching: A collaborative multi-centre ESHRE study. Hum. Reprod..

[19] Van Rij M.C., De Rademaeker M., Moutou C., Dreesen J.C., De Rycke M., Liebaers I., Geraedts J.P., De Die-Smulders C.E., Viville S. (2012). Preimplantation genetic diagnosis (PGD) for Huntington’s disease: The experience of three European centres. Eur. J. Hum. Genet..

[20] Shenfield F., Pennings G., Devroey P., Sureau C., Tarlatzis B., Cohen J. (2003). Taskforce 5: Preimplantation genetic diagnosis. Hum. Reprod..

[21] Smeets B., Sallevelt S.C.E.H., Dreesen J.C., De Die-Smulders C.E., De Coo I., Die-Smulders C.E. (2015). Preventing the transmission of mitochondrial DNA disorders using prenatal or preimplantation genetic diagnosis. Ann. N. Y. Acad. Sci..

[22] Berckmoes V., Verdyck P., De Becker P., De Vos A., Verheyen G., Van Der Niepen P., Verpoest W., Liebaers I., Bonduelle M., Keymolen K. (2019). Factors influencing the clinical outcome of preimplantation genetic testing for polycystic kidney disease. Hum. Reprod..

[23] Ubaldi F.M., Capalbo A., Vaiarelli A., Cimadomo D., Colamaria S., Alviggi C., Trabucco E., Venturella R., Vajta G., Rienzi L. (2016). Follicular versus luteal phase ovarian stimulation during the same menstrual cycle (DuoStim) in a reduced ovarian reserve population results in a similar euploid blastocyst formation rate: New insight in ovarian reserve exploitation. Fertil. Steril..

[24] Chamayou S., Sicali M., Alecci C., Ragolia C., Liprino A., Nibali D., Storaci G., Cardea A., Guglielmino A. (2017). The accumulation of vitrified oocytes is a strategy to increase the number of euploid available blastocysts for transfer after preimplantation genetic testing. J. Assist. Reprod. Genet..

[25] Hu X., Ding C., Zhang D., Zhou W., Wang J., Zeng Y., Lv J., Xu Y., Zhou C.-Q. (2017). Embryo pooling: A promising strategy for managing insufficient number of embryos in preimplantation genetic diagnosis. Gynecol. Endocrinol..

[26] De Vos A., Staessen C., De Rycke M., Verpoest W., Haentjens P., Devroey P., Liebaers I., Van de Velde H. (2009). Impact of cleavage-stage embryo biopsy in view of PGD on human blastocyst implantation: A prospective cohort of single embryo transfers. Hum. Reprod..

[27] De Rycke M., Goossens V., Kokkali G., Meijer-Hoogeveen M., Coonen E., Moutou C. (2017). ESHRE PGD Consortium data collection XIV-XV: Cycles from January 2011 to December 2012 with pregnancy follow-up to October 2013. Hum. Reprod..

[28] Cimadomo D., Rienzi L., Capalbo A., Rubio C., Innocenti F., García-Pascual C.M., Ubaldi F.M., Handyside A. (2020). The dawn of the future: 30 years from the first biopsy of a human embryo. The detailed history of an ongoing revolution. Hum. Reprod. Update.

[29] Scott R.T., Upham K.M., Forman E.J., Zhao T., Treff N.R. (2013). Cleavage-stage biopsy significantly impairs human embryonic implantation potential while blastocyst biopsy does not: A randomized and paired clinical trial. Fertil. Steril..

[30] Zakharova E.E., Zaletova V.V., Krivokharchenko A.S. (2014). Biopsy of Human Morula-Stage Embryos: Outcome of 215 IVF/ICSI Cycles with PGS. PLoS ONE.

[31] Irani M., Zaninovic N., Canon C., O’Neill C., Gunnala V., Zhan Q., Palermo G., Reichman D., Rosenwaks Z. (2018). A rationale for biopsying embryos reaching the morula stage on Day 6 in women undergoing preimplantation genetic testing for aneuploidy. Hum. Reprod..

[32] Magli M.C., Albanese C., Crippa A., Tabanelli C., Ferraretti A.P., Gianaroli L. (2019). Deoxyribonucleic acid detection in blastocoelic fluid: A new predictor of embryo ploidy and viable pregnancy. Fertil. Steril..

[33] Van Landuyt L., Polyzos N.P., De Munck N., Blockeel C., Van de Velde H., Verheyen G. (2015). A prospective randomized controlled trial investigating the effect of artificial shrinkage (collapse) on the implantation potential of vitrified blastocysts. Hum. Reprod..

[34] Brouillet S., Martinez G., Coutton C., Hamamah S., Sophie B., Guillaume M., Charles C., Samir H. (2020). Is cell-free DNA in spent embryo culture medium an alternative to embryo biopsy for preimplantation genetic testing? A systematic review. Reprod. Biomed. Online.

[35] Capalbo A., Romanelli V., Patassini C., Poli M., Girardi L., Giancani A., Stoppa M., Cimadomo D., Ubaldi F.M., Rienzi L. (2018). Diagnostic efficacy of blastocoel fluid and spent media as sources of DNA for preimplantation genetic testing in standard clinical conditions. Fertil. Steril..

[36] Leaver M., Wells D. (2019). Non-invasive preimplantation genetic testing (niPGT): The next revolution in reproductive genetics?. Hum. Reprod. Update.

[37] Loutradi K.E., Kolibianakis E., Venetis C.A., Papanikolaou E.G., Pados G., Bontis I., Tarlatzis B.C. (2008). Cryopreservation of human embryos by vitrification or slow freezing: A systematic review and meta-analysis. Fertil. Steril..

[38] Bosch E., De Vos M., Humaidan P. (2020). The Future of Cryopreservation in Assisted Reproductive Technologies. Front. Endocrinol..

[39] Rechitsky S., Ström C., Verlinsky O., Amet T., Ivakhnenko V., Kukharenko V., Kuliev A., Verlinsky Y. (1999). Accuracy of Preimplantation Diagnosis of Single-Gene Disorders by Polar Body Analysis of Oocytes. J. Assist. Reprod. Genet..

[40] Spits C., De Rycke M., Verpoest W., Lissens W., Van Steirteghem A., Liebaers I., Sermon K. (2006). Preimplantation genetic diagnosis for Marfan syndrome. Fertil. Steril..

[41] Coskun S., Alsmadi O. (2007). Whole genome amplification from a single cell: A new era for preimplantation genetic diagnosis. Prenat. Diagn..

[42] Spits C., Le Caignec C., De Rycke M., Van Haute L., Van Steirteghem A., Liebaers I., Sermon K. (2006). Whole-genome multiple displacement amplification from single cells. Nat. Protoc..

[43] Langmore J.P. (2002). Rubicon Genomics, Inc. Pharmacogenomics.

[44] Zong C., Lu S., Chapman A.R., Xie X.S. (2012). Genome-Wide Detection of Single-Nucleotide and Copy-Number Variations of a Single Human Cell. Science.

[45] Deleye L., Coninck D.D., Christodoulou C., Sante T., Dheedene A., Heindryckx B., Abbeel E.V.D., Sutter P.D., Menten B., Deforce D. (2015). Whole genome amplification with SurePlex results in better copy number alteration detection using sequencing data compared to the MALBAC method. Sci. Rep..

[46] De Bourcy C.F.A., Vlaminck I.D., Kanbar J.N., Wang J., Gawad C., Quake S.R. (2014). A Quantitative Comparison of Single-Cell Whole Genome Amplification Methods. PLoS ONE.

[47] Deleye L., Gansemans Y., De Coninck D., Van Nieuwerburgh F., Deforce D. (2018). Massively parallel sequencing of micro-manipulated cells targeting a comprehensive panel of disease-causing genes: A comparative evaluation of upstream whole-genome amplification methods. PLoS ONE.

[48] Renwick P., Trussler J., Lashwood A., Braude P., Ogilvie C.M. (2010). Preimplantation genetic haplotyping: 127 diagnostic cycles demonstrating a robust, efficient alternative to direct mutation testing on single cells. Reprod. Biomed. Online.

[49] LaFramboise T. (2009). Single nucleotide polymorphism arrays: A decade of biological, computational and technological advances. Nucleic Acids Res..

[50] Handyside A.H., Harton G.L., Mariani B., Thornhill A.R., Affara N., Shaw M.-A., Griffin D.K. (2009). Karyomapping: A universal method for genome wide analysis of genetic disease based on mapping crossovers between parental haplotypes. J. Med. Genet..

[51] García-Bermúdez M., Piyamongkol W., Tomaz S., Dudman E., Sherlock J.K., Wells D. (2003). Single-cell sequencing and mini-sequencing for preimplantation genetic diagnosis. Prenat. Diagn..

[52] Chow J.F.C., Cheng H.H.Y., Lau E.Y.L., Yeung W.S.B., Ng E.H.Y. (2020). Distinguishing between carrier and noncarrier embryos with the use of long-read sequencing in preimplantation genetic testing for reciprocal translocations. Genomics.

[53] Kubicek D., Hornak M., Horak J., Navratil R., Tauwinklova G., Rubes J., Vesela K. (2019). Incidence and origin of meiotic whole and segmental chromosomal aneuploidies detected by karyomapping. Reprod. Biomed. Online.

[54] Yin X., Tan K., Vajta G., Jiang H., Tan Y., Zhang C., Chen F., Chen S., Zhang C., Pan X. (2013). Massively Parallel Sequencing for Chromosomal Abnormality Testing in Trophectoderm Cells of Human Blastocysts1. Boil. Reprod..

[55] Yan L., Huang L., Xu L., Huang J., Ma F., Zhu X., Tang Y., Liu M., Lian Y., Liu P. (2015). Live births after simultaneous avoidance of monogenic diseases and chromosome abnormality by next-generation sequencing with linkage analyses. Proc. Natl. Acad. Sci. USA.

[56] Chamayou S., Sicali M., Lombardo D., Alecci C., Ragolia C., Maglia E., Liprino A., Cardea C., Storaci G., Romano S. (2019). Universal strategy for preimplantation genetic testing for cystic fibrosis based on next generation sequencing. J. Assist. Reprod. Genet..

[57] Del Rey J., Vidal F., Ramírez L., Borràs N., Corrales I., Garcia I., Garcia-Martínez I., Fernandez S.F., Garcia-Cruz R., Pujol A. (2018). Novel Double Factor PGT strategy analyzing blastocyst stage embryos in a single NGS procedure. PLoS ONE.

[58] De Geyter C., Calhaz-Jorge C., Kupka M.S., Wyns C., Mocanu E., Motrenko T., Scaravelli G., Smeenk J., Vidakovic S., Goossens V. (2020). ART in Europe, 2015: Results generated from European registries by ESHRE. Hum. Reprod. Open.

[59] De Rycke M., De Vos A., Belva F., Berckmoes V., Bonduelle M., Buysse A., Keymolen K., Liebaers I., Nekkebroeck J., Verdyck P. (2020). Preimplantation genetic testing with HLA matching: From counseling to birth and beyond. J. Hum. Genet..

[60] Davies M.J., Moore V.M., Willson K.J., Van Essen P., Priest K., Scott H., Haan E.A., Chan A. (2012). Reproductive Technologies and the Risk of Birth Defects. Obstet. Gynecol. Surv..

[61] Pandey S., Shetty A., Hamilton M., Bhattacharya S., Maheshwari A. (2012). Obstetric and perinatal outcomes in singleton pregnancies resulting from IVF/ICSI: A systematic review and meta-analysis. Hum. Reprod. Update.

[62] Zhu J., Zhu Q., Wang Y., Wang B., Lyu Q., Kuang Y. (2018). Comparative study on risk for birth defects among infants after in vitro fertilization and intracytoplasmic sperm injection. Syst. Boil. Reprod. Med..

[63] Desmyttere S., De Rycke M., De Schrijver F., Verpoest W., Haentjens P., Staessen C., Liebaers I., Bonduelle M. (2011). Neonatal follow-up of 995 consecutively born children after embryo biopsy for PGD. Hum. Reprod..

[64] Bay B., Ingerslev H.J., Lemmen J.G., Degn B., Rasmussen I.A., Kesmodel U.S. (2016). Preimplantation genetic diagnosis: A national multicenter obstetric and neonatal follow-up study. Fertil. Steril..

[65] Heijligers M., Van Montfoort A., Meijer-Hoogeveen M., Broekmans F., Bouman K., Homminga I., Dreesen J., Paulussen A., Engelen J., Coonen E. (2018). Perinatal follow-up of children born after preimplantation genetic diagnosis between 1995 and 2014. J. Assist. Reprod. Genet..

[66] He H., Jing S., Lu C.F., Tan Y.Q., Luo K.L., Zhang S.P., Gong F., Lu G.X., Lin G. (2019). Neonatal outcomes of live births after blastocyst biopsy in preimplantation genetic testing cycles: A follow-up of 1721 children. Fertil. Steril..

[67] Winter C., Van Acker F., Bonduelle M., Desmyttere S., De Schrijver F., Nekkebroeck J. (2014). Cognitive and psychomotor development of 5- to 6-year-old singletons born after PGD: A prospective case-controlled matched study. Hum. Reprod..

[68] Winter C., Van Acker F., Bonduelle M., Desmyttere S., Nekkebroeck J. (2015). Psychosocial development of full term singletons, born after preimplantation genetic diagnosis (PGD) at preschool age and family functioning: A prospective case-controlled study and multi-informant approach. Hum. Reprod..

[69] Sacks G.C., Altarescu G., Guedalia J., Varshaver I., Gilboa T., Levy-Lahad E., Eldar-Geva T. (2016). Developmental neuropsychological assessment of 4- to 5-year-old children born following Preimplantation Genetic Diagnosis (PGD): A pilot study. Child Neuropsychol..

[70] Heijligers M., Peeters A., Van Montfoort A., Nijsten J., Janssen E., Gunnewiek F.K., De Rooy R., Van Golde R., Coonen E., Meijer-Hoogeveen M. (2019). Growth, health, and motor development of 5-year-old children born after preimplantation genetic diagnosis. Fertil. Steril..

[71] Belva F., Roelants M., Kluijfhout S., Winter C., De Schrijver F., Desmyttere S., De Rycke M., Tournaye H., Liebaers I., Bonduelle M. (2018). Body composition and blood pressure in 6-year-old singletons born after pre-implantation genetic testing for monogenic and structural chromosomal aberrations: A matched cohort study. Hum. Reprod. Open.

